# Levatiracetam for the management of Lance-Adams syndrome

**Published:** 2014

**Authors:** Faik ILIK, Mustafa Kemal ILIK, İlker ÇÖVEN

**Affiliations:** 1 Department of Neurology, Elbistan State Hospital, Kahramanmaras, Turkey; 2 Department of Neurosurgery, Faculty of Medicine, Mevlana University, Konya, Turkey; 3 Department of Neurosurgery, Faculty of Medicine, Başkent University, Konya, Turkey

**Keywords:** Lance-Adams syndrome, Levatiracetam, Post-hypoxic myoclonus

## Abstract

Chronic post-hypoxic myoclonus, also known as Lance-Adams syndrome (LAS) is a neurological complication characterized by uncontrolled myoclonic jerks following cardiac arrest. In this article, clinical manifestation and symptomatic treatment options are discussed especially concerning the rationale of use of levatiracetam in patients with Lance-Adams syndrome. Clinical presentation is action myoclonus associated with cerebellar ataxia, postural imbalance, and very mild intellectual deficit.

An 18-year-old female patient was admitted to our intensive care unit in a coma. She had a cardiorespiratory arrest after a splenectomy in a local hospital.

Then, myoclonic movements were continuously observed over the entire body, including the face.

On day 14 of hospitalization, we started levatiracetam 1000 mg daily. The frequency of convulsion movements was reduced. The patient level of consciousness was 15 on the Glasgow coma scale (GCS) on the Mini-Mental State Examination (MMSE) score was 23 out of 30. She was later transferred to

the rehabilitation department.

Vigilance is required to ensure early diagnosis and timely intervention for the myoclonic jerks. We would like to emphasize that LAS should be considered in patients with the myoclonic jerks following cardiac arrest and that levatiracetam therapy may be useful as treatment.

## Introduction

Post-hypoxic myoclonus (PHM) is a neurological complication characterized by uncontrolled myoclonic jerks following cardiac arrest. Two types of PHM, acute and chronic, occur in patients with hypoxic injury of the brain. Chronic PHM, also known as Lance-Adams syndrome (LAS), first described in 1963, is a condition characterized by development of chronic post-hypoxic action myoclonus due to a temporary lack of or inadequate brain oxygen supply (1-2). Clinical presentation is action myoclonus associated with cerebellar ataxia, postural imbalance, and very mild intellectual deficit (3). In this report, we present a case of Lance-Adams syndrome and discuss its main clinical manifestation and symptomatic treatment options of this rare disease in the light of the relevant literature.

## Case Study

**Fig 1:**
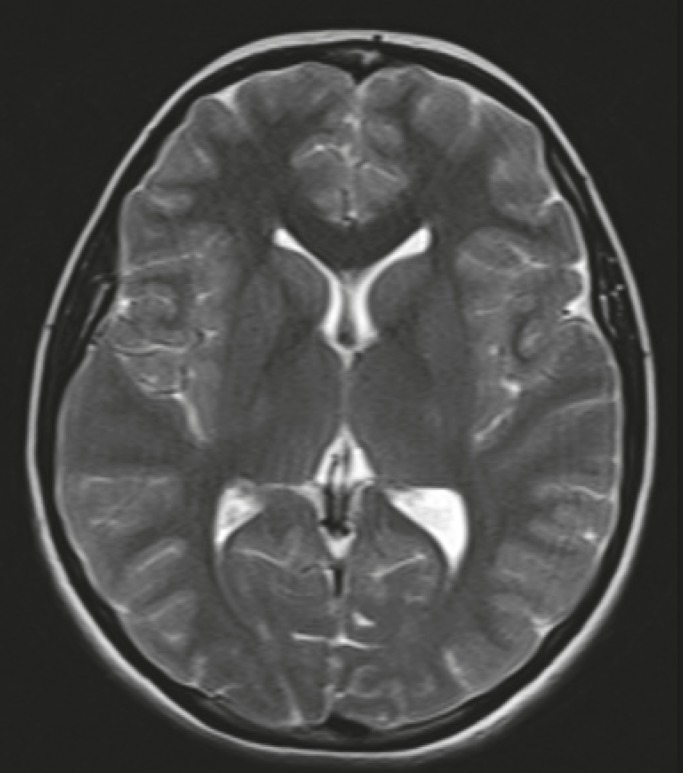
MRI showed no cerebral abnormality

An 18-year-old female patient was admitted to our intensive care unit in a coma. The past medical history of the patient was normal without any additional problems. She had a cardiorespiratory arrest after a splenectomy in a local hospital. On day 3 of hospitalization, she developed a generalized seizure in the intensive care unit after discontinuing an intravenous infusion of midazolam (5 mg per hour) and fentanyl (100 μg). Then, myoclonic movements were continuously observed over the entire body, including the face. Cranial computed tomography (CT) scans, T1 and T2 weighted MR imaging, and DW imaging showed no abnormality (Fig 1). Seven days later, the patient regained her consciousness and had no hemiparesis or aphasia. Sodium valproate and clonazepam were started; however, the myoclonic jerks were not controlled after increasing the dose of sodium valproate to 1,500 mg and clonazepam to 3 mg daily. 

**Fig 2:**
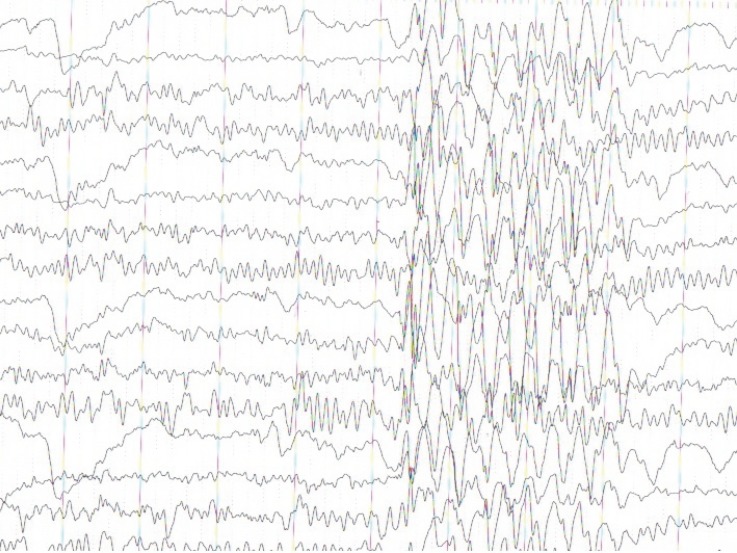
EEG showed generalized paroxysmal epileptic activity

AN electroencephalography (EEG) performed at this time demonstrated frequent myoclonic polyspikes but did not confirm myoclonic status (Fig 2). She continued to have a moderate generalized myoclonus in the face, trunk, and limbs accompanied by dysmetria, dysarthria, and ataxia. Generalized myoclonic jerks were aggravated by voluntary movements, sounds, and touches, but disappeared with a relaxation of the body or during a sleep. On day 14 of hospitalization, we started levatiracetam 1000 mg daily. The frequency of convulsion movements was reduced. Her level of consciousness was 15 on the Glasgow coma scale (GCS). Her Mini-Mental State Examination (MMSE) score was 23 out of 30. On day 27 of hospitalization, she was transferred to the rehabilitation department. 

## Discussion

Lance-Adams syndrome (LAS) was first described in the 1960s by Lance and Adams. Clinical presentation is action myoclonus associated with cerebellar ataxia, postural imbalance, and very mild intellectual deficit.

Myoclonic jerks are specifically triggered by action and tactile stimulation. They usually disappear with relaxation of the body. Diagnostic imaging tests such as CT or MRI of the brain are not helpful to make a diagnosis of LAS. Neuroimaging, such as brain singlephoton emission computed tomography (SPECT) or brain positron emission tomography (PET) has recently shown anatomical and pathophysiological basis of LAS (5). Although, the pathophysiology of LAS has not been clearly defined, the prognosis is known to be quite good (4). On day 3 of hospitalization, our patient from the case- study developed a myoclonic jerk after cardiorespiratory arrest. Generalized myoclonic jerks were aggravated by voluntary movements, sounds, and touches, but disappeared with a relaxation of the body and limbs or during a sleep. She had mild cognitive deficit accompanied by dysmetria and dysarthria. These clinical features fulfilled the criteria for the diagnosis of Lance-Adams-type myoclonus. The myoclonus in LAS has no consistent correlation with EEG abnormalities. A combination of medications based on the neurotransmitter hypotheses is often needed to obtain appropriate control of symptoms. Frucht and Fahn reviewed more than 100 patients with LAS, they found that clonazepam, valproate, and piracetam were effective in treating approximately 50% of these cases(6). The patient underwent consecutive trials of sodium valproate to 1,500 mg daily and clonazepam to 3 mg daily without efficacy. We began with 1000 mg levatiracetam daily nd the myoclonic jerks terminated on the 8th day of treatment. Clonazepam and valproate were gradually discontinued. Further studies to clarify the pathogenesis of LAS are important to find novel therapeutic methods.

The neurotransmitters related to LAS are known to be serotonin and gamma-aminobutyric acid (GABA). The loss of serotonin within the inferior olive nucleus has been thought to play some role and GABA may interact with the serotonin system to suppress post-hypoxic myoclonus (7).

Vigilance is required to ensure early diagnosis and timely intervention for the myoclonic jerks. 

**In conclusion,** we would like to emphasize that LAS should be considered in patients with the myoclonic jerks following cardiac arrest and that levatiracetam therapy may be useful as treatment.
